# A smart scaffold composed of three-dimensional printing and electrospinning techniques and its application in rat abdominal wall defects

**DOI:** 10.1186/s13287-020-02042-6

**Published:** 2020-12-10

**Authors:** Zhi Yang, Zhicheng Song, Xin Nie, Kaijin Guo, Yan Gu

**Affiliations:** 1 Department of Orthopedics, Affiliated Hospital of Xuzhou Medical University, Xuzhou, 221002 China; 2 Department of General Surgery, Shanghai Ninth People’s Hospital Affiliated to Shanghai Jiao Tong University School of Medicine, Hernia and Abdominal Wall Surgery Center of Shanghai Jiao Tong University, Shanghai, 200011 China

**Keywords:** Abdominal wall defect, Three-dimensional print, Electrospinning, Tissue engineering, Myogenesis

## Abstract

**Background:**

Biological composite scaffolds are increasingly being used in abdominal wall reconstruction but still have certain shortcomings. The present study describes here a novel three-dimensional (3D) scaffold fabricated by combining 3D printing (3DP) and electrospinning (ESP).

**Methods:**

Biological composite scaffolds are composed of integrated 3DP interconnected macrofiber and random ESP microfiber networks. The 3DP scaffold retains intact 3D architecture and mechanical properties, while the ESP network serves as a cell entrapment system at the extracellular matrix (ECM) scale. Biological composite scaffolds are implanted in a defective rat abdominal wall to detect if it could induce early vascularization and reconstruction of the tissue defect.

**Results:**

SEM analysis reveals a pore diameter of 424.47 ± 58.49 μm and a porosity of 70.46 ± 2.48% for biological composite scaffolds. In the in vitro test of cell proliferation, biological composite scaffolds do not affect rat dermal fibroblast proliferation in a time- and dose-dependent manner. The animal experiments show tissue remodeling and early angiogenesis as compared to 3DP scaffolds.

**Conclusions:**

Our experiment prepares a biological scaffold with both a macro- and microscale structure by ESP and 3DP technology. Thus, the integration of 3DP and ESP techniques provides a new set of smart scaffolds for abdominal wall defect and hernia repair.

**Supplementary Information:**

The online version contains supplementary material available at 10.1186/s13287-020-02042-6.

## Introduction

Abdominal wall reconstruction is one of the most common surgical procedures. Abdominal defects typically occur following traumas, hernia formation, and excision after primary or metastatic neoplasm. Among them, reconstruction following major, complex abdominal wall defects is a significant clinical challenge. Tension-free repair techniques using implantable materials are the gold standard for treating these problems because a direct primary suture results in a high rate of recurrence [1–3]. Among these implantable materials, scaffolds, which are biocompatible, biodegradable, structurally stable, and hydrophilic, were used to repair or replace damaged tissue in tissue engineering and regenerative medicine [4, 5]. Previously, scaffolds were fabricated using traditional techniques such as gas foaming, fiber bonding, solution casting, melt molding, freeze-drying, and gas expansion [6, 7]. These methods enable easy fabrication of three-dimensional (3D) scaffolds; however, controlling the scaffold pore size and porosity is difficult [8, 9]. Moreover, scaffolds manufactured using these methods do not have fully interconnected pores. Three-dimensional printing (3DP) is a promising approach for engineering biomaterial scaffolds characterized by custom-shaped and fully interconnected networks of pore [10]. It is often used in designing and fabricating hard tissue scaffolds such as bones [11]. However, scant research exists on fabricating soft tissue scaffolds using 3DP technology.

The mechanical properties of a tissue scaffold depend on the suitability of the structural materials. Soft elastomers make 3DP scaffolds more like soft tissue. There are a few safe and biodegradable homopolymers applicable in the domain of 3DP such as l-lactide (LLA), glycolide, and ε-caprolactone. Among them, the homopolymer of caprolactone, poly (ε-caprolactone) (PCL), is a flexible, tough semicrystalline polymer with an elongation at break of more than 400% at room temperature [9]. Poly (l-lactide) (PLLA), on the other hand, is a semicrystalline polymer with high stiffness and low elongation at break [12]. Copolymerization of l-lactide and ε-caprolactone (PLC) is an effective method to produce soft materials with properties intermediate to those of the parent homopolymers.

However, cell-seeding efficiency and tissue formation remain limited by the scaffolds’ pore resolution. The pore size of 3DP scaffolds is relatively large compared to the size of a cell. As a result, multiple cells are required to obtain enough adherent cells that can produce sufficient extracellular matrix (ECM) to successfully engineer tissue construction. Aggregating the cells prior to seeding may improve cell-seeding efficiency, because cell populations are more likely to be trapped in the pores [10]. By enhancing the cell–cell signaling, cell aggregation results in better tissue formation. Another solution is introducing micron-sized fiber networks into the 3DP scaffolds. This has the dual advantage of acting as a “sieve” for cell entrapment while providing ECM-like niches to the cells [13]. Such a multi-scale network can be created by combining 3DP with another novel technology called electrospinning (ESP). The dimensions of electrospun (ESP) fibers typically vary from the nano- to the microscale, owing to the application of a high-voltage electric field to a polymeric solution pumped into the field. The diameter and surface topologies of the fibers vary according to the electric field intensity, pumping flow rate, and polymer solution concentration. In addition, the size and surface texture of the ESP scaffolds have been shown to influence the rate and morphology of cell proliferation [14].

Therefore, the aim of this study was to examine the feasibility of 3DP technique of PLC material with electrospinning using collagen I which was applicable in the soft tissue scaffolds. Then, the 3DP scaffolds with electrospinning (biological composite scaffolds) and the mere 3DP scaffolds were observed through a series of in vitro and in vivo experiments to assess the influence of the microfibrillar network on cell entrapment and proliferation, mechanical property, and tissue remodeling. Of these, the positive control group, porcine small intestine submucosa (PSIS)—a clinically used scaffold, would be selected for the in vivo portion of the experiments to further compare their performance in the rat abdominal wall defect model.

## Materials and methods

### Substrates and solvents

ε-Caprolactone (99%) was purchased from Aladdin Reagent Co. (Shanghai, China), dried over CaH_2_, and distilled under nitrogen at reduced pressure. l-Lactide, collagen type I, and stannous octoate (SnOct_2_) (95%) were purchased from Sigma-Aldrich (St. Louis, MO, USA), and 1,4-butanediol (99%) was purchased from Shenke Biotechnology Co. (Shanghai, China). Anhydrous toluene (99.8%) was purchased from Aldrich, dried over CaH_2_, and distilled under nitrogen.

### Polymer synthesis

The PLC copolymer was polymerized using 50 mol% ε-caprolactone and 50 mol% l-lactide. In addition, 0.03 mol% SnOct_2_ and 0.05 mol% 1,4-butanediol of the total monomer amount were used. Ring-opening batch polymerization was performed at 160 °C for 4 h. The copolymer was purified by dissolving it in dichloromethane, filtering the resultant solution, and precipitating the polymer with ethanol.

### Nuclear magnetic resonance spectrometry (NMR)

^13^C NMR spectra were obtained using a Bruker 400 spectrometer, with CDCl_3_ as the internal standard. The samples were dissolved in deuterated chloroform in 5-mm-diameter sample tubes. One-milliliter solvent contained 100 mg of polymer.

### Scaffold fabrication

Biological composite scaffolds were fabricated by combining a Bioplotter device (Envisiontec GmbH, Germany) with a home-made ESP device. The Bioplotter is originally an XYZ plotter device, as previously described [12]. The polymers were briefly placed in a stainless-steel syringe, subsequently heated at 190 °C by a thermostatic cartridge device, and fixed on the “X”-moving arm of the device. When the molten stage was reached, a nitrogen pressure of 5 bar was utilized to the syringe through a pressurized cap. Rectangular block models were loaded on the Bioplotter CAM software (PrimCAM, Switzerland) and deposited layer by layer by extrusion of the polymer as a fiber. The deposition speed was set to 300 mm/min. The scaffolds were then characterized by the fiber diameter, spacing between the fibers in the same layer, layer thickness, and configuration of the deposited fibers within the whole architecture. In our study, the inner diameter of the nozzle was set to 400 μm, the layer thickness was set to 225 μm, the fiber spacing was set to 800 μm, and the scaffold structure was defined by a 0–90 layer configuration in which fibers were deposited in 90° oriented steps between successive layers.

ESP fibrous networks were produced from a 20% w/v polymer solution in a 90%/10% v/v chloroform/hexafluoroisopropanol mixture. The ESP device consisted of a high-voltage (0–30 kV) generator (NCE 30000, Heinzinger Electronic GmbH, Germany) connected to a collector plate and a syringe containing the polymer solution. When a high voltage was used, an electric field was formed between the syringe needle (positive pole) and collector (negative pole). The polymer solution was then pumped out of the syringe at variable flow rates and pulled through the electrostatic field to form a jet filament. Dry fibers were randomly deposited on the collector. The fibrous network was characterized by the applied voltage, air gap (distance between the syringe needle and collector plate), pump flow rate, syringe needle, and polymer solvent used. In our experimental setup, the voltage was kept constant at 15 kV, the air gap was fixed at 15 cm, the flow rate was 0.39 ml/min, and the inner diameter of the needle used was 0.9 mm.

The biological composite scaffolds were fabricated by electrospinning a fibrous network layer on alternate layers of 3D fiber-deposited mesh until the scaffold height became 4 mm. The influence on cell entrapment and tissue formation was evaluated using two different network densities. The ESP fiber density was determined by the time frame used in the electrospinning process.

### Scanning electron microscopy (SEM)

Using a Philips-XL-30 instrument (FEI, Hillsboro, OR, USA), the surface morphology of the 3DP scaffolds and 3DP with ESP collagen film scaffolds was analyzed by SEM. The samples were fixed in 2% glutaraldehyde for 2 h at 4 °C, washed twice in phosphate buffer solution (PBS), and post-fixed in 1% osmic acid for 2 h at 4 °C. After two PBS washes, the samples were dehydrated in a graded series of ethanol, dried to a critical point (HCP-2; Hitachi, Tokyo, Japan), mounted, sputter-coated with gold (BAL-TEC; FEI), and analyzed by SEM.

### Animals

Sixty Sprague-Dawley rats weighing 200–250 g were purchased from SLAC National Rodent Laboratory Animal Resources (Shanghai, China). Of these, a total of fifty-four male rats were randomly divided into three groups (*n* = 18 per group) and implanted with 3DP, biological composite scaffolds, or PSIS. The rest of the rats were used for in vitro experiments. All the animal experiments were approved by the Institutional Review Committee of Shanghai Jiao Tong University School of Medicine (ID: SYXK 2008-0050). The environment for these animals was maintained at 18–26 °C, with a relative humidity of 30–60%.

### Cell culture and seeding

Rat dermal fibroblasts (RDFs) were isolated from the rat dermis and cultured in Dulbecco’s modified Eagle’s medium (Sigma-Aldrich), supplemented with 10% fetal bovine serum (Invitrogen) and 1% penicillin/streptomycin at 37 °C in a 5% CO_2_ incubator. The cells used in this study were not passaged more than four times. 3DP scaffolds, both with and without ESP collagen film, were cut into 1 × 1 cm^2^ sheets and washed twice in the PBS. The RDFs were resuspended in culture medium at 1 × 10^6^/ml and uniformly seeded onto these scaffolds; 10 ml of culture medium was gently added after the scaffolds were placed in a 37 °C incubator for 4 h. The medium was replaced every 2 days. The incubation of the cell-loaded scaffolds depended on further cellular experiments.

### Cell proliferation

The 3DP and biological composite scaffolds were soaked in 75% alcohol for 1 h, sterilized by UV irradiation for 1 h, and then, the extracts were prepared according to the ratio of scaffold weight to extract delivery (DMEM culture medium containing 10% fetal bovine serum) of 1 mg:1 ml, and incubated in a CO^2^ incubator for 48 h. Second-generation rat fibroblasts (2 × 10^4^/l) were grown on 96-well plates, and the blank group was a simple DMEM culture medium containing 10% fetal bovine serum. The cell number was analyzed on days 1, 3, 5, and 7 with the Cell Counting Kit-8 (Dojindo Laboratories). The optical density (OD) of each well was measured by enzyme-linked immunoassay at a wavelength of 450 nm, and the cytotoxicity of each group was compared according to the OD value.

### Tissue repair in full-thickness abdominal wall defect model

In total, 54 male rats, which were randomly divided into three groups (*n* = 18 per group) and implanted with PSIS, 3DP, or biological composite scaffolds, were used. The rats were anesthetized using an intraperitoneal injection of 10% chloral hydrate (4 ml/kg). A longitudinal midline skin incision (3 cm) was made, and a full-thickness abdominal wall defect (3 × 2 cm^2^), including the fascia, underlying rectus abdominis muscle, and peritoneum, was created. The scaffold was placed intra-abdominally with a 0.3-cm overlap and fixed tension-free to the abdominal wall using an interrupted, non-resorbable 5–0 monofilament nylon suture (Tianqing Biological Material Co. Ltd., Shanghai, China). The skin incision was sutured using absorbable 2–0 Vicryl (Ethicon, Somerville, NJ, USA), and the rats were allowed to recover normally. These animals were checked every week for local and systemic complications such as incision dehiscence, wound infection, intestinal fistula, bulge, or recurrent hernia.

### Macroscopic examination

Six rats each from the 2- and 4-week post-implantation groups and all the rats from the 12-week post-implantation group were euthanized for further experiment. Meanwhile, the animals were examined for any evidence of hematoma, seroma, or implant infection on the subcutaneous and visceral sides. Two surgeons who were blinded to the research independently scored the intraperitoneal adhesions and percentage of implants covered by adhesions according to the previously described criteria [15]. The thickness of the implant was measured in five random samples from the central part of the implant.

### Histological examination

The implants were dissected from the repaired part and horizontal interface of the surrounding tissues and defect area. The samples were fixed in 4% paraformaldehyde, following which they were embedded in paraffin, cut into 5-μm-thick sections, and subjected to Masson’s trichrome staining and immunohistochemical labeling with antibodies against cluster of differentiation CD15 (1:200; Santa Cruz Biotechnology, Santa Cruz, CA, USA) and CD31 (1:200; Millipore, Billerica, MA, USA).

Further, two pathologists who were blinded to the specimens being tested qualitatively analyzed the samples independently. The analysis included an assessment of the extent and type of cellular infiltration and the presence and degree of neovascularization. The number of infiltrated inflammatory cells was determined by numbering the immunopositive cells in six matched microscope fields at × 400 magnification (E600; Nikon, Tokyo, Japan). The neovascularization was assessed based on the immunohistochemical detection of CD31-positive endothelial cells, and a blood vessel was defined as a luminal structure with or without red blood cells. Similarly, the number of blood vessels was counted in six matched microscope fields at × 200 magnification. The tissue areas in the microscope images were calculated from the scale bar.

### Biomechanical analysis

The biomechanics of the 50 × 10 mm^2^ samples, including the normal abdominal wall, were calculated using an Instron Model 5542 uniaxial material testing machine (Canton, Norwood, MA, USA). The samples were immersed in 100-ml PBS at 37 °C for 1 h. Then, the two ends of the sample were held between 25-mm-apart grippers. The samples were gradually stretched by increasing the distance between the grippers at a cross-head speed of 10 mm/min until failure. The load (N) and displacement (mm) were recorded throughout the elongation and converted to a stress–strain curve, with respect to the initial sample dimensions. Five samples of each material were tested.

### Statistical analysis

The data were expressed as mean ± SD. The PSIS, 3DP, or biological composite scaffold results were compared using the one-way analysis of variance test. The SPSS v.13.0 software (SPSS Inc., Chicago, IL, USA) was used for statistical analysis. *P* < 0.05 was considered statistically significant.

## Results

### Scaffold characterization

**Fig. 1:**
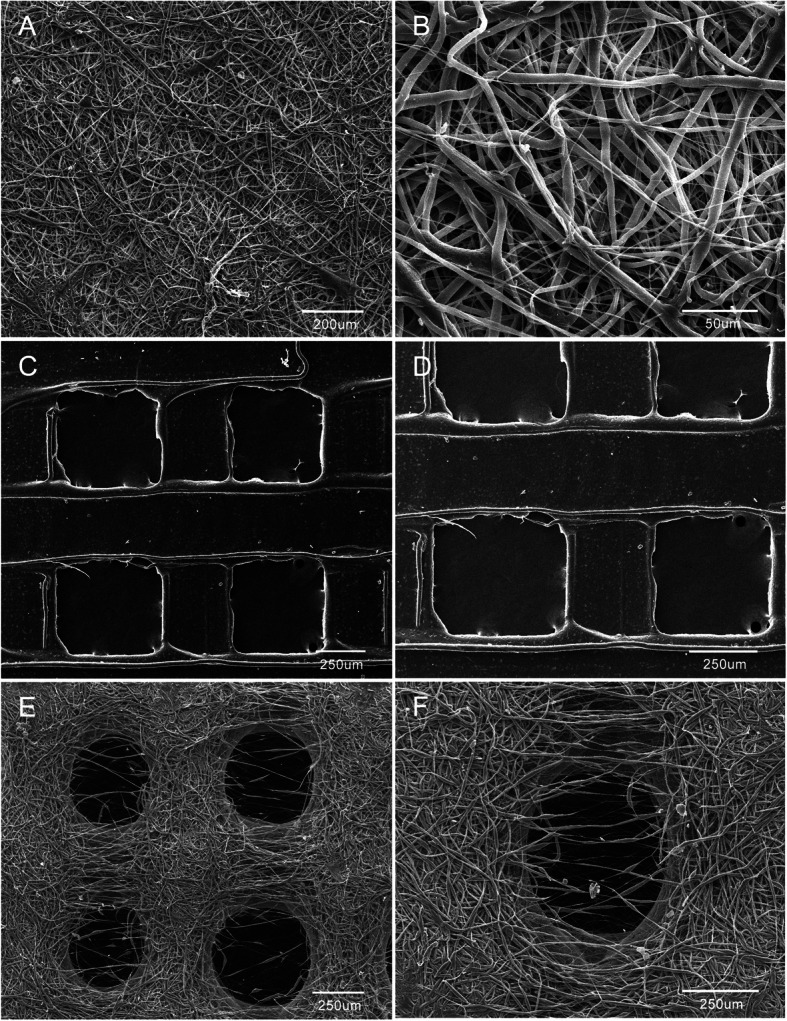
Representative scanning electron micrographs of collagen I nanofiber (**a**, **b**), 3DP (**c**, **d**), and biological composite scaffold (**e**, **f**) samples

The results of the SEM analysis of the collagen I nanofiber membranes, 3DP, and biological composite scaffolds are shown in Fig. 1. The diameters of the nanofiber membranes varied from hundreds of nanometers to several micrometers. The SEM analysis revealed a pore diameter of 424.47 ± 58.49 μm for the 3DP scaffolds and 392.17 ± 36.06 μm for the biological composite scaffolds. This corresponded to a porosity of 73.56 ± 1.06% and 70.46 ± 2.48% for the 3DP scaffolds and biological composite scaffolds, respectively. There were no differences in the pore diameter and porosity statistics.

### In vitro test of cell proliferation in 3D scaffolds

**Fig. 2:**
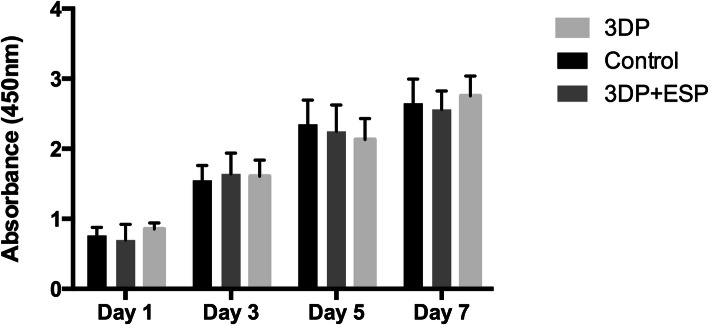
The fibroblast toxicity comparison of 3DP and biological composite scaffolds

In the proliferation assessment (Fig. 2), the absorbance values were similar across groups at each time point. At the end of the proliferation period, there were no significant differences among these three groups, which indicated that PLC material and electrospun collagen I fibers are not significantly toxic to the proliferation of rat fibroblasts.

### Macroscopic observations

**Fig. 3:**
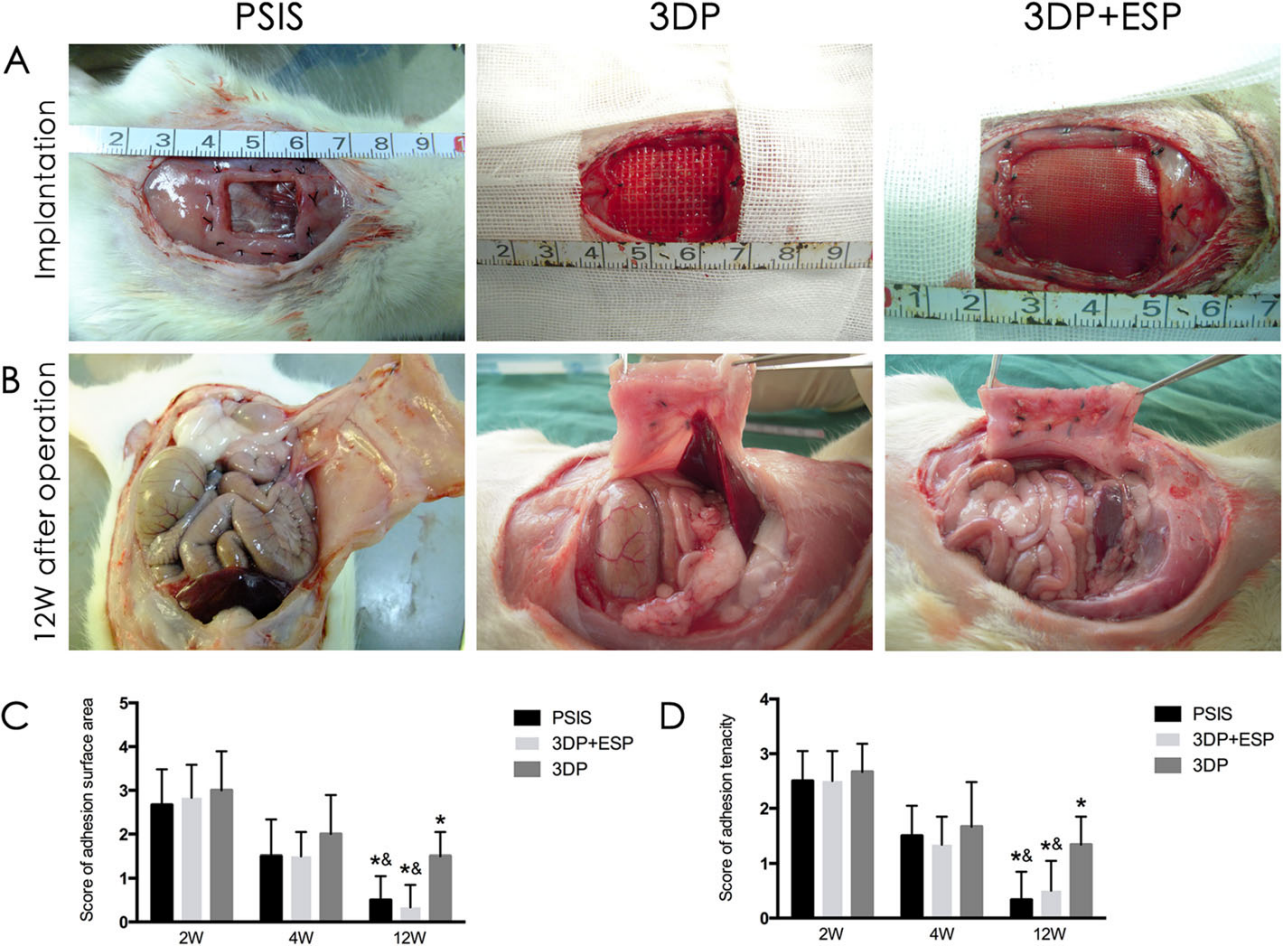
Macroscopic observation and adhesion condition assessment of PSIS, 3DP, and biological composite scaffolds. *P < 0.05 vs. 2W within a group, ^#^*P* vs. 1M within a group, ^&^*P* < 0.05 vs. the 3DP group in the same time point

During the early postoperative period (< 2 weeks post-surgery), four patients in the PSIS group and two in the 3DP group developed seromas that were spontaneously resorbed. In three patients in the 3DP group and one biological composite scaffold case, wound infections that showed no signs of dehiscence and did not require any intervention occurred. None of the rats showed evidence of bulging or herniation at the implantation site until sacrifice. Three months later, the color of the surgical site in rats of the biological composite scaffold group was still pale, compared to the peripheral muscle tissue (Fig. 3). However, there was no clear boundary in the PSIS group.

### Inflammatory response, neovascularization, and new collagen deposition

**Fig. 4:**
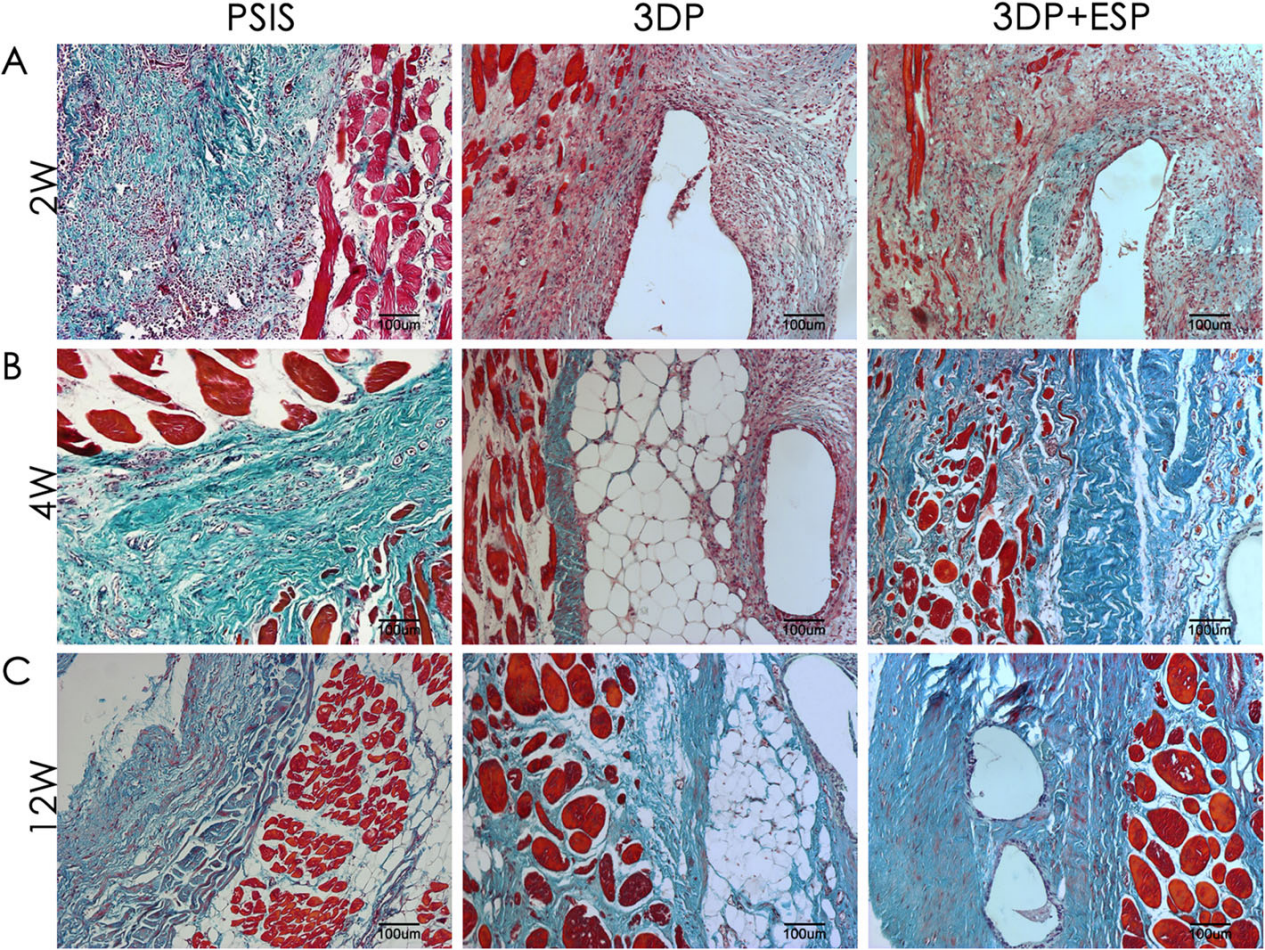
Masson’s trichrome staining of PSIS, 3DP, and biological composite scaffolds during repair of an abdominal wall defect 2 weeks (2W), 1 month (1M), and 3 months (3M) after implantation

**Fig. 5:**
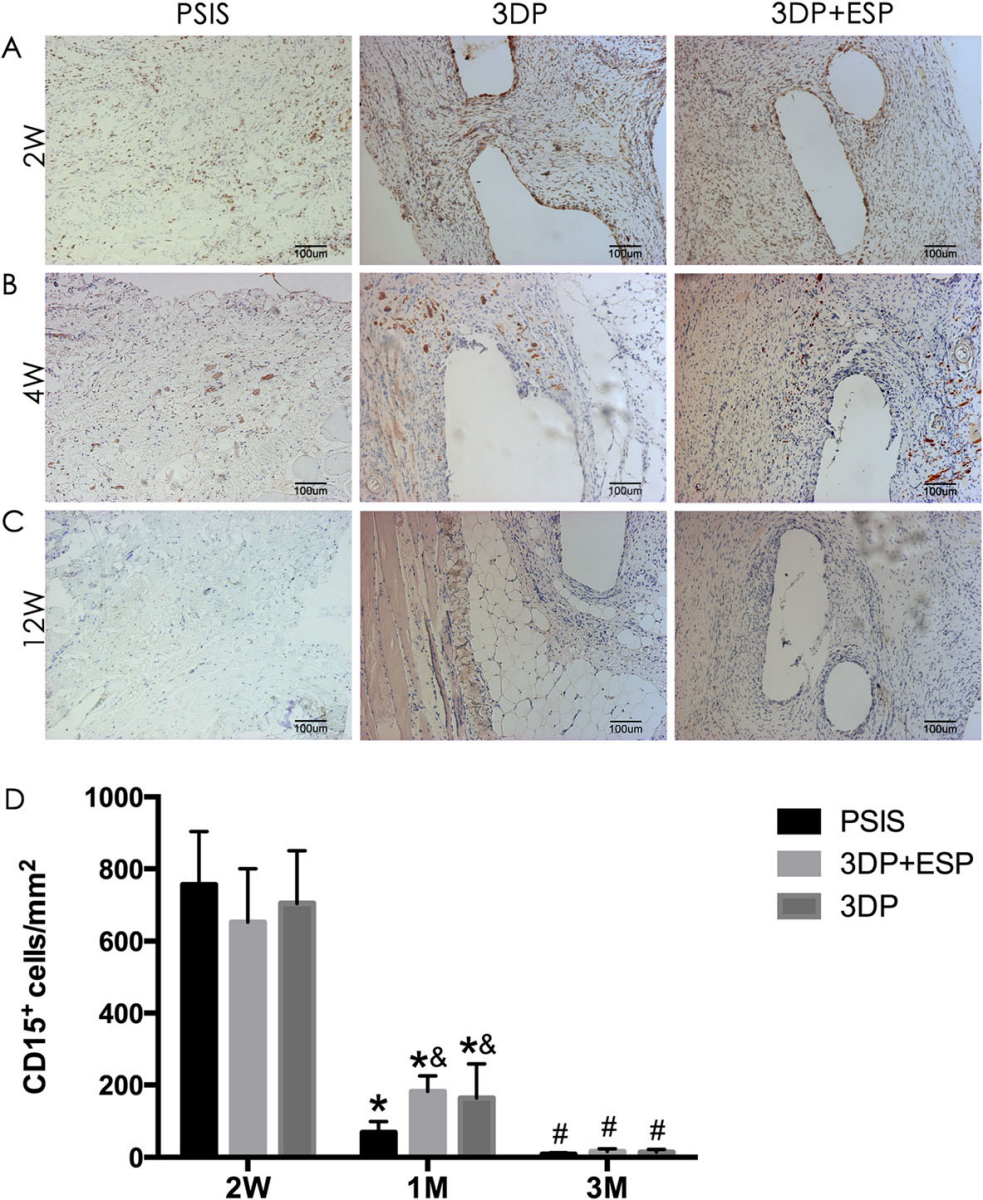
Immunohistochemical detection of CD15 expression in abdominal wall defects 2 weeks (2W), 1 month (1M), and 3 months (3M) after implantation of PSIS, 3DP, and biological composite scaffolds. Quantitative analysis of CD15-positive cells 2 weeks (2W), 1 month (1M), and 3 months (3M) after implantation of PSIS, 3DP, and biological composite scaffolds. *P < 0.05 vs. 2W within a group, ^#^*P* vs. 1M within a group, ^&^*P* < 0.05 vs. the PSIS group in the same time point

**Fig. 6:**
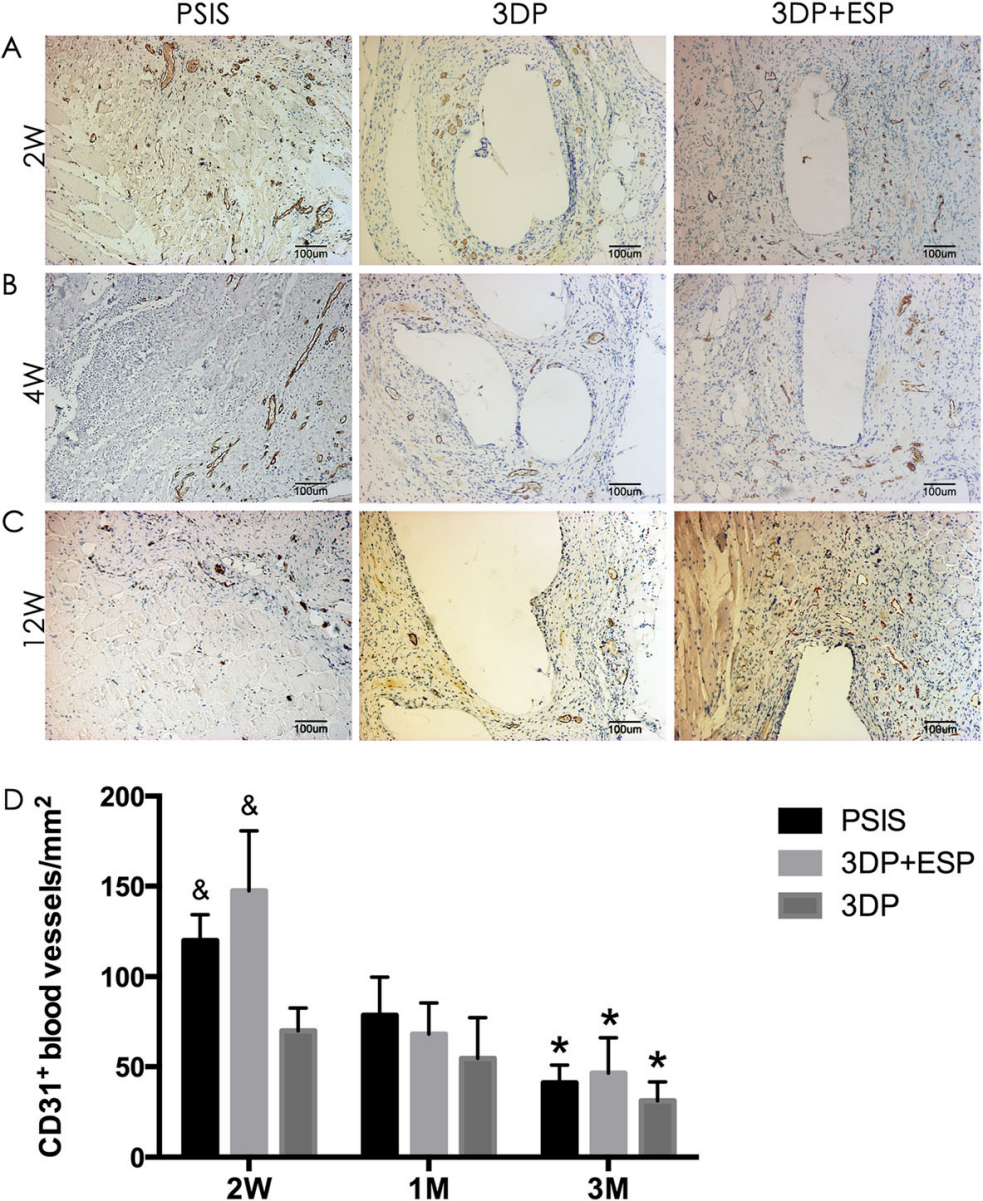
Immunohistochemical detection of CD31-positive blood vessels in PSIS, 3DP, and biological composite implants 2 weeks (2W), 1 month (1M), and 3 months (3M) after implantation. Quantitative analysis of CD31-positive blood vessels 2 weeks (2W), 1 month (1M), and 3 months (3M) after implantation of PSIS, 3DP, and biological composite scaffolds. *P < 0.05 vs. 2W within a group, ^&^*P* < 0.05 vs. the 3DP group in the same time point

The PSIS, 3DP, and biological composite scaffolds were evaluated 2, 4, and 12 weeks after implantation through histological staining. At 2 weeks, Masson’s trichrome staining revealed new collagen fiber deposition around these three scaffolds (Fig. 4a), with more extensive cellular infiltration in the PSIS and biological composite scaffold groups. Immunohistochemistry revealed the presence of CD15-positive granulocytes (Fig. 5a) on the border of and inside these three types of scaffolds. There were more CD31-positive blood vessels in the PSIS and biological composite scaffold groups than in the 3DP group (*P* < 0.05) (Fig. 6a, d).

The collagen fibers were better aligned in these groups at 4 weeks, than at previous time points (Fig. 4b). The PSIS degraded more quickly than the 3DP and biological composite scaffolds. The number of granulocytes reduced in all the groups (Fig. 5b, d), especially in the PSIS group (*P* < 0.05). Interestingly, the level of neovascularization decreased dramatically in the PSIS and biological composite scaffold groups (*P* < 0.05) (Fig. 6b, d).

At 12 weeks post-implantation, the PSIS implants had degraded almost completely, whereas the 3DP and biological composite implants were still present, as indicated by Masson’s trichrome staining (Fig. 4c). Moreover, the adipose tissue was more distributed around and within the 3DP scaffold, compared to the PSIS scaffold. The granulocytes were rare in all the groups until the latest time point (Fig. 5c, d). The groups contained similar number of CD31-positive blood vessels (Fig. 6c, d).

### Biomechanical properties

**Fig. 7:**
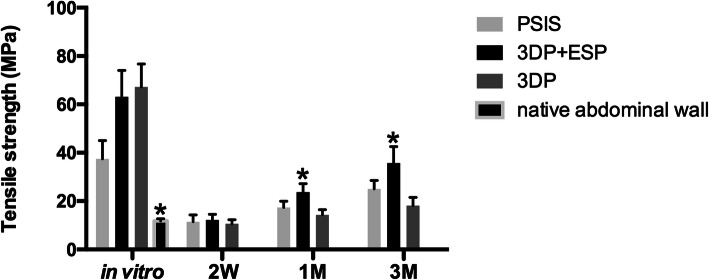
The in vitro tensile strength of native abdominal wall, PSIS, 3DP, and biological composite scaffolds and in vivo strength of incorporation were measured. *P < 0.05 vs. any other groups in vitro and in vivo

The tensile strength of the PSIS, 3DP, and biological composite samples was greater than that of the native abdominal wall (*P* < 0.05) in vitro (Fig. 7). The in vivo biomechanical properties of PSIS, 3DP, and biological composite implants were examined after 2, 4, and 12 weeks. The tensile strength of these three types of scaffolds decreased rapidly at 2 weeks, with no significant difference among the three (Fig. 7), and increased steadily thereafter. At the final time point, the value was higher in the biological composite samples than in the 3DP and PSIS samples (*P* < 0.05), which is different from the in vitro results.

## Discussion

A variety of acellular biological scaffolds, such as the PSIS, pericardial membrane, and acellular dermal matrix, have been used for abdominal wall reconstruction as well as other soft tissue repair because of the limitations associated with synthetic meshes in many clinical settings [5, 16–18]. However, there has been interest in identifying more desirable biological scaffolds. In the present study, the biocompatibility, biomechanical, and morphological properties of 3DP and biological composite scaffolds were investigated before and after implantation. Notably, the abdominal wall tissues of rats repaired with the biological composite scaffolds were better than those repaired with the PSIS.

Generally, collagen is widely used as an ideal material for tissue regeneration, as collagen, the main structural element of ECM, can alter cell morphology, migration, and, in some cases, differentiation [19, 20]. However, in spite of its enormous potential as a biomaterial, its application for soft tissue regeneration has been restricted by low mechanical properties. To overcome the shortcomings of collagen, we proposed a hybrid process (an ESP-based rapid-prototyping method) to fabricate biological composite scaffolds, consisting of vertical PLC struts that provide mechanical support to the scaffolds, and collagen fibers that promote the bioactivity of the scaffolds. In this study, the fabricated hybrid scaffolds showed satisfactory mechanical tensile properties. Furthermore, compared to pure PLC 3DP scaffolds, they provide a biocompatible, supportive environment for recellularization and vascularization following implantation.

In vivo study was examined whether biological composite scaffold could act as a repair and provide adequate support for large abdominal wall defects. Recurrent hernia is one of the most serious complications after reconstruction of large abdominal wall defects. Although no clinical recurrence was observed in either group, macroscopic observation suggested that biological composite meshes occurred with greater integration in host tissues compared to 3DP scaffolds. Implant adhesion is another important indicator of abdominal wall repair success, and severe visceral adhesions can induce complications such as intestinal perforation and bowel obstruction. The biological composite meshes and 3DP, both absorbable biological meshes, resulted in minimal intra-abdominal adhesion.

Host responses were different in the biological composite and 3DP groups. Although inflammatory reactions elicited by the biological meshes were initially (at 2 weeks) similar for both groups, as evidenced by the presence of granulocytes, the location of the inflammatory cells varied. The cells were diffusely distributed in the biological composite group, whereas they were localized at the border area in the 3DP group. At the final time point, the inflammatory response was abated in the biological composite and 3DP groups. The initial incorporation of the 3DP implant into the host tissue was slower compared to the biological composite implant, as evidenced by a lower collagen deposition and capillary growth. However, after peaking at 4 weeks, neovascularization began to decrease with both types of implants. This could be due to many factors, most notably the acute inflammatory response and implant characteristics [21, 22]. As the implants degraded during remodeling and consolidated in the healing process, the inflammatory stimulus of the formed fibrous connective tissue diminished, resulting in reduced levels of neovascularization later in the repair process.

Although the in vitro tensile strength was greater in the biological composite and 3DP scaffolds than in the PSIS scaffolds, the converse was observed following implantation. The reported mechanical data for a material do not represent their actual strength, but rather the degree of integration between the graft and adjacent host tissue, i.e., the strength of incorporation [23–25]. During mechanical testing, most implants tore at the interface. The strength of the incorporation with the surrounding tissues initially decreased and then, over time, gradually increased; thus, due to the inferior integration of the 3DP scaffolds, their in vivo tensile strength was lower at the latest time point.

Although we attempted to provide an exhaustive description of the biological composite scaffolds, our study still has some limitations. First, although the data on comparison were acquired up to 3 months post-implantation, additional studies over a longer period of time are required to further evaluate the mechanical changes, scaffold remodeling, and myogenesis. Second, the PSIS implants used in this study are representative of commercially available crosslinked scaffolds, which are widely used in clinical setting. But our results suggest that crosslinking may delay the integration and repair efficiency of host tissues. Therefore, future studies will include a comparison of biological composite and non-crosslinked implant types, which may provide additional clinically relevant data on the effectiveness of the biological composite scaffolds.

## Conclusion

This study is the examination of the in vitro properties of the biological composite scaffold and comparison of the structural and functional remodeling of the biological composite scaffold and PSIS implants. The results demonstrate that the biological composite scaffold retained intact 3D architecture and mechanical properties, whereas the ESP network served as a cell entrapment system on the ECM scale. When implanted in a rat’s defective abdominal wall, the biological composite scaffold could induce early vascularization and exhibited the ability to remodel the tissue defect. These findings indicated that biological composites were promising biomaterials for abdominal wall and hernia repair.

## Supplementary Information

**Additional file 1.**

**Additional file 2.**

## Data Availability

All data generated or analyzed during this study are included in this published article.

## Abbreviations

PSIS: Porcine small intestine submucosa
UBM: Urinary bladder matrix
3DP: Three-dimensional printing
ESP: Electrospinning
ECM: Extracellular matrix
LLA: l-Lactide
PCL: Poly (ε-caprolactone)
PLLA: Poly (l-lactide)
NMR: Nuclear magnetic resonance spectrometry
SEM: Scanning electron microscope
RDFs: Rat dermal fibroblasts
DMEM: Dulbecco’s modified Eagle’s medium
